# Liquid harvesting and transport on multiscaled curvatures

**DOI:** 10.1073/pnas.2011935117

**Published:** 2020-09-08

**Authors:** Chuxin Li, Cunlong Yu, Shan Zhou, Zhichao Dong, Lei Jiang

**Affiliations:** ^a^Chinese Academy of Sciences Key Laboratory of Bio-inspired Materials and Interface Sciences, Technical Institute of Physics and Chemistry, Chinese Academy of Sciences, 100190 Beijing, China;; ^b^School of Future Technology, University of Chinese Academy of Sciences, 101407 Beijing, China

**Keywords:** directional liquid transport, biomimetic structure, multiscaled curvature, fog harvest

## Abstract

Various creatures, such as spider silk and cacti, have harnessed their surface structures to collect fog for survival. These surfaces typically stay dry and have a large contact hysteresis enabling them to move a condensed water droplet, resulting in an intermittent transport state and a relatively reduced speed. In contrast to these creatures, here we demonstrate that *Nepenthes alata* offers a remarkably integrated system on its peristome surface to harvest water continuously in a humid environment. Multicurvature structures are equipped on the peristome to collect and transport water continuously in three steps: nucleation of droplets on the ratchet teeth, self-pumping of water collection that steadily increases by the concavity, and transport of the acquired water to overflow the whole arch channel of the peristome. The water-wetted peristome surface can further enhance the water transport speed by ∼300 times. The biomimetic design expands the application fields in water and organic fogs gathering to the evaporation tower, laboratory, kitchen, and chemical industry.

## Significance

Although various creatures possess different structural gradients to gather water from fog, such as spider silk and cactus cluster, their gradient surfaces can only drive the motion of harvested water droplets over only a limited drop-sized distance and afford a slow speed, limiting the practical usage. The peristome of the pitcher plant presented here is a superior fog harvestor that capitalizes on the combined effects of ratchet, concavity, and arch structures to collect water from humid air and transport condensate water directionally along the curved peristome at a recording speed. Biomimetic approaches apply this multiscaled curvatures design to the construction of water fog and organic vapor harvestor, making it a versatile solution for a broad range of applications.

Fresh water is rapidly becoming the most precious commodity which sustains human life and is vital for health. The shortage of fresh water is one of the most pressing global issues (1). Water scarcity affects more than 40% of the global population and is projected to rise, especially in some of the world’s most impoverished countries (2). The demand of fresh water is expected to increase constantly due to the population growth (3), for example, farmers almost drain a giant inland lake in Central Asia to achieve enough agricultural water for farming (4). Developing new water source shall be of benefit to resolving the freshwater shortage issue (5, 6).

Fog is a valuable freshwater source necessary for the survival of plants and insects, especially in arid regions (7–10). Various natural creatures possess unique structural features that exhibit controlled dropwise condensation and transport properties (11–13). Intriguing water-harvesting examples include the conical spines of the *Cactus* cluster (7), periodic spindle knots and joints of spider silk (8), multiscale structured hair of *Syntrichia caninervis* (9), and trichome of *Sarracenia* (10). These creatures harvest water when fog comes. The surface energy gradient or structural gradient that exists on these natural surfaces plays a vital role in water gathering (7–10). Laplace pressure induced by these gradient structures drives the motion of harvested water droplets on their dry surfaces, but only at a limited distance, typically of the size of a single drop (7, 8, 12, 14). This condition leads to a severe problem in practical usage: the droplets are transported along the gradient and need to merge into large ones and then slide or drip down the mesh, wire, or substrate (15–19). Sliding a gathered drop on a titled substrate requires the threshold drop radius (15) to be above a large size of [(cos*θ*_r_ − cos*θ*_a_)/sin*α*]^1/2^*l*_c_, where *l*_c_ is the capillary length and equals (*γ*/*ρg*)^1/2^, *θ*_r_ is the receding contact angle (CA), and *θ*_a_ is the advancing CA. The onset of shedding limits drop collection efficiency. It is essential to design surfaces that enable droplets to grow rapidly and to be shed as quickly as possible (19).

Nature offers a remarkably integrated system to help *Nepenthes alata* survive in a nutrient-poor habitat with high humidity (13, 20–22): condensing water from humid air and transporting it along the peristome to construct a slippery aqueous layer (13) that is used for sliding insects into the trap to enable the plant to digest sources of nitrogen (22, 23). Numerous studies have been performed on the *Nepenthes* species and demonstrate the role of microscaled concavities arrays or arch structures in controlling water directional transport (24–28). The mechanisms for water collection and transport have never been completely revealed.

Here, we reveal the generation mechanism of the slippery aqueous layer on the peristome of the *N. alata* plant and demonstrate the multicurvature-enhanced superior water harvest and transport abilities. The peristome capitalizes on the surface curvatures present on the conical teeth (ratchet), concavities, and arch channels to harvest and transport water condensation continuously and quickly. Even though the teeth tips are pointed downward (22), the ratchet teeth and concavities can enhance the Laplace pressure, resulting in the antigravity transport of water condensation. Curvatures between neighboring teeth work synergistically to prevent the dripping of condensate water and successfully push the collected water upward in an ultrafast speed. The formation of the water layer on the peristome further enhances the subsequent water-harvesting speed. A recording water harvesting and transport speed is achieved by the combined effect of ratchet, concavity, and arch structures. We apply this methodology to harvest and transport water, isopropanol, kerosene, glycol, and gasoline fog on various artificial materials, including polyvinyl alcohol (PVA) and polydimethylsiloxane (PDMS), and show that the harvest and transport efficiency is preserved even in harsh operating environments. This multiscaled curvature design, inspired by water-harvest strategies in pitcher plants, would innovate the construction of water and organic vapor harvestor and expand the scope of application in evaporation tower, chemical industry, laboratory, and even in kitchen.

## Results

### Multicurvature Structures of Natural Peristome Surface.

**Fig. 1.**
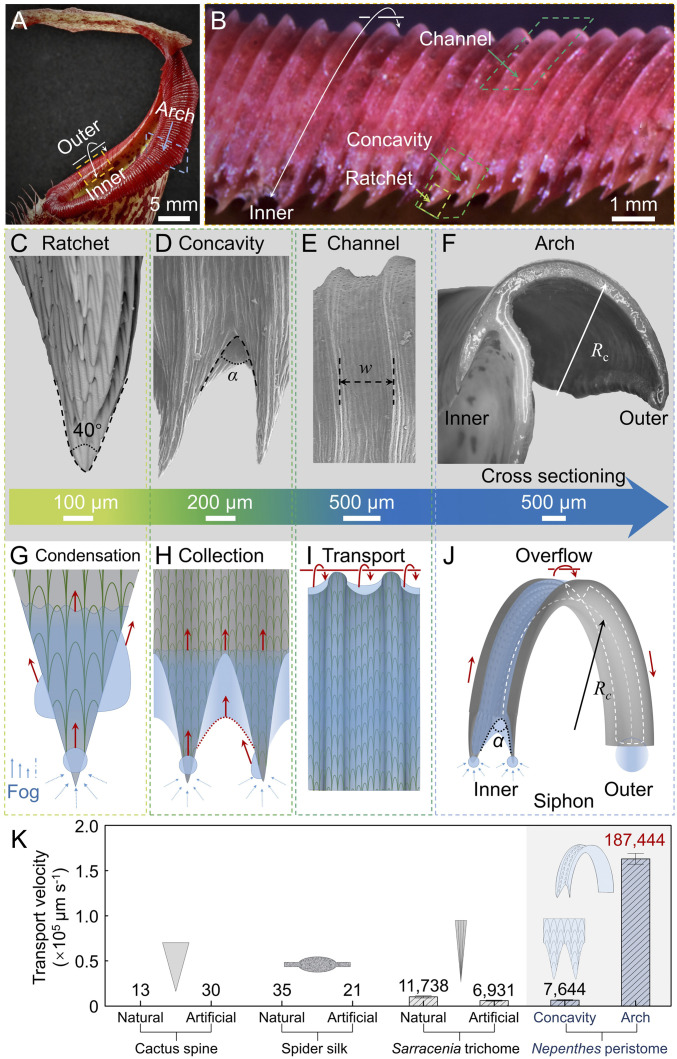
Sketch of water condensation and transport process on peristome. (*A*) Optical image of the *N. alata* pitcher. (*B*) Inner view of the ratchet teeth projections along the inner edge of the peristome. The ratchet-teeth tips point downward. (*C*–*E*) The feature structures of the peristome, including the ratchet, concavity, and channel. (*C*) Straight rows of overlapping epidermal cells with tips toward the opposite direction of the teeth projections. (*D*) The concavity in between neighboring teeth. (*F*) Cross-sectioning of the arch-shaped peristome. (*G*–*J*) Schematic images of water condensation and transport on the peristome. Water condenses on the ratchet teeth first and then transports upward to the concavity, which acts as both a collector and a pump to deliver water until it overflows the arch-shaped channel to the outer rim. The communicating vessels constructed after wetting speed up the water-harvesting efficiency. (*K*) Transport velocity of water condensation on different natural and artificial structures in the dry state for Cactus spine and spider silk and in the wet state for the *Sarracenia* trichome. The concavity of the peristome can greatly enhance the water transport speed in the wet state. The global water transport velocity on the concavity is 7,644 ± 766 μm s^−1^. The combined effects of ratchet, concavity, and arch accelerate the water transport speed to 187,444 ± 6,814 μm s^−1^. See *SI Appendix*, Table S1 for details.

Mature *N. alata* plants bear extensively modified “pitchers” at the tips of leaves that are capable of attracting, trapping, and digesting insects and absorbing the released nutrients (29, 30). At the upper rim of the pitcher, *N. alata* typically has a conspicuous prey-trapping peristome (Fig. 1*A*). The peristome is an arch-shaped and double-edged collar (Fig. 1*B*) with a diameter ranging from 20 to 35 mm along the collar plane and a width of 5–10 mm from the inner to the outer side (22). The detailed surface morphology of the peristome surface in a zooming state is shown in Movie S1. Teeth projections grow along the inner edge of the peristome (31). Since a pitcher typically grows in the vertical direction (30), most of the ratchet teeth face downward (Fig. 1*B*). Scanning electron microscope (SEM) images reveal the detailed morphology of the ratchet teeth along the inner edge of the peristome (Fig. 1 *C* and *D*). Each tooth has a conical shape, with a cap at the narrower tip with a diameter of 45.7 ± 2.3 μm and a width of 628.5 ± 7.5 μm at the base. The concavity in between neighboring teeth forms a concave structure, the opening angle, *α*, of which is 45° (Fig. 1*D*). Above the ratchet or concavity, the peristome is composed of highly directionally organized ridges with hierarchical structures (13). The hierarchical structures that derive from the tips of the ratchet teeth form two ordered structures that are regularly distributed along the peristome surface (Fig. 1*E*). Neighboring larger ridges form channels with a width equal to that between teeth (23). Within larger ridges, duck-billed microcavities are repeatedly arrayed, which form smaller-order ridges (13). Submillimeter-scaled and micrometer-scaled cavity structures are arranged on the curved peristome surface from the inner side to the outer side, forming an arch channel; the radius of curvature of the arch-shaped peristome is ∼2.3 mm (Fig. 1*F*). The ratchet, concavity, and arch channel structures with different curvatures form peristome surface features (Fig. 1 *A*–*F*).

In this work, we focus on the role of peristome feature structures, namely, the ratchet teeth, concavities, and arch channel structures, in controlling water condensation and transport (Fig. 1 *G*–*J*). Schematic images in Fig. 1 show how the peristome is a superior water collection system consisting of hierarchical curvatures that work together to harvest and transport water effectively. The detailed mechanism is demonstrated in *Discussion*. The accelerated refresh (Movie S2) of water nucleation sites at the ratchet teeth is induced by the synergistic transport effect of the Laplace pressure induced by the conical ratchets and the suction force at the concavities (Fig. 1 *G* and *H*). The accumulated water in the concavities spontaneously climbs the peristome surface in the vertical direction and then overflows at the arch to form a thin water layer (Fig. 1 *I* and *J*). The water transport velocity on the concavity of the peristome is 7,644 ± 766 μm s^−1^, more than 200 times faster than that on the cactus spine and spider silk (Fig. 1*H*). The self-constructed wet surface can even increase the water-harvesting efficiency after “communicating vessels” are formed by the arch channel. Considering a small droplet has a larger Laplace pressure than a larger drop, the small droplet at the ratchet side will empty into the larger drop gathered at the other side of the arch. The combined effect induced by the ratchet, concavity, and arch curvatures drives the water motion from the inner side to the outer side and increases the transport speed (Fig. 1*J*). The water transport velocity on the arch channel of natural peristome surface is 187,444 μm s^−1^, about tens of times faster than the water transport velocity on *Sarracenia* trichome and tens of thousands times of that on the cactus spine and spider silk, respectively (Fig. 1*K*).

## Discussion

### Fog Harvesting and Transport Processes along Peristome Surface.

**Fig. 2.**
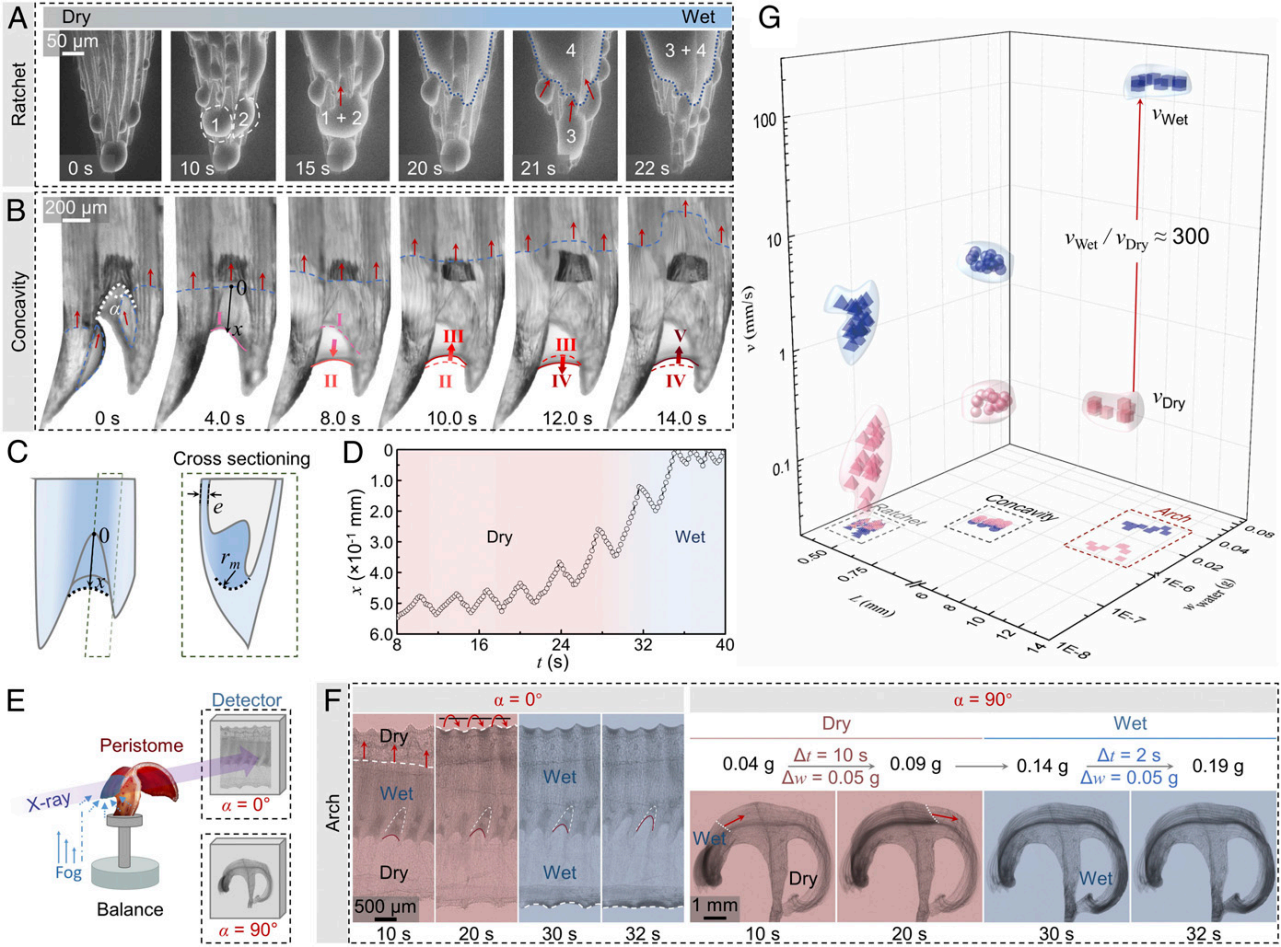
Water condensation and transport processes on natural peristome. (*A*) Water droplets are first condensed on the dry ratchet teeth and collected together to form larger droplets. After water droplets merge or climb up, the ratchet teeth surface becomes wet. Droplets at the dry–wet boundary slide into the wet region quickly. The tip is refreshed, with the water being transported upward. (*B*) Droplets climb the ratchet teeth and merge into a larger drop at the concavity. The pump of water from state I to state II and back to state III forces water to climb upward successively. The red arrows indicate the transport direction of the water droplets and the blue dotted line marks the wet–dry dividend line. (*C*) Schematic image of the pump mechanism. Laplace pressure forces condensate water to flow upward. (*D*) Plots of the water meniscus distance (*x*) from the concavity tip measured in *B* as a function of time (*t*). Water gathered in the concavity reduces as the dry peristome becomes wet. (*E*) The experimental setup for the X-ray observation. The stage rotation facilitates the observation from orthogonal views. A high-sensitivity balance system records the water-gathering weight during the condensation process. (*F*) The transport of water condensation eventually results in the development of a thin water film that covers the arch-shaped peristome surface. To help locate the positions of the dry–wet boundary line, the red arrows and white dashed lines indicate the parts of the surface that are eventually filled with water. (*G*) Individual condensate droplet weight with corresponding transport speed as a function of the condensation surface particular length on the dry and wet peristome surfaces. Condensate water transport on the wet peristome surface has a 300 times higher speed than that of water transport on the dry surface.

Environmental scanning electron microscopy (ESEM), high-speed cameras, and X-ray imaging techniques are used to investigate the water condensation–transport phenomena (Fig. 2). The time-sequence images shown in Fig. 2 are divided into three magnifications that focus on the action of the ratchet teeth (Fig. 2*A*), concavities (Fig. 2*B*), and arch channels (Fig. 2*F*). ESEM is used to monitor the water condensation state on the ratchet teeth (Fig. 2*A*). When the peristome is exposed to water fog, water condenses on the teeth. The cone structure (14) can facilitate condensate water transport away from that location to refresh the interface. The condensate water drops 1 and 2 merge into 1 + 2 and grow in size (32) and climb the dry structures at a speed of 1.36 × 10^−1^ mm s^−1^. Continuous condensate water can wet the cone structure. Comparing with dry structures, the wet structures can wick the merged water drop 3 from the cone to the teeth base at a faster speed of 2.25 mm s^−1^. The wet cone structure can accelerate the transport speed at 16.5 times. Besides water condensation, the ratchet teeth capture all available water droplets, such as raindrops (*SI Appendix*, Fig. S1).

High-speed camera records the water transport dynamics. When the dry peristome surface is just placed in fog at a relative humidity of 95%, condensate droplets on the neighboring ratchet teeth generally do not coalesce with each other but simply move individually to the base, as the liquid front indicated by the arrows in Fig. 2*B*. As the condensate droplets grows in size, the neighboring droplets then combine into a large droplet at the concavity. A capillary bridge connects either side of the cone structures. The meniscus grows as the deposition proceeds, from state I to state II in Fig. 2*B*, introducing a more significant pressure gradient across the wetting pattern (33, 34). Considering water menisci pins at a distance of *x* from the center with the menisci radius, *r*_m_, in the cross-sectioning (Fig. 2*C* and *SI Appendix*, Fig. S2), the Laplace pressure is regarded as Eq. 1:

$$\Delta P=\gamma \left(\frac{1}{r_{m}}-\frac{\cos\theta }{\alpha x}\right),$$ [1]

where *γ* is the water surface tension, *θ* is the water contact angle, and *α* is the opening angle of the concavity, respectively. As the wetting states change from II to III, the expulsive force induced by the capillary effect can cause the gathered water to flow upward in a pulsed fashion. A new cycle with transitions from III to V “pumps” the water along the peristome surface repeatedly. The variation of *x* decreases as water covers the whole peristome surface, indicating a low pump force is needed to overcome the resistance (Fig. 2*D*). In addition, the maximum water-climbing height, *H*_max_, supported by the concavity can be regarded as Eq. 2:

$$H_{\max}=l_{c}^{2}\left(\frac{1}{r_{m}}-\frac{\cos\theta }{\alpha x}\right),$$ [2]

where *l*_*c*_ is the capillary length and equals (*γ*/*ρg*)^1/2^. *H*_max_ is ∼60 mm. Considering the radius of the peristome is only 2.3 mm, the *H*_max_ is enough for water to cover the whole peristome surface (13, 20, 25). The covering water film can steadily remain on the peristome without leakage (Movie S2).

The whole water-harvesting process over a long duration is recorded by X-ray imaging and high-sensitivity balance system. Fig. 2*E* provides in-line projection images of the system. The rotation of the sample stage enables the system to record the process from the front- and cross-sectioning views, respectively. The weight change of 5.0 × 10^−2^ g, recorded by the balance, requires 10 s in a dry state, whereas it requires only 2 s when the peristome is wet (Fig. 2*F*). As the water film forms on the peristome after condensation, the subsequent droplets can wick along the wet surface from one side to the other with a lower resistance. The correlation between the timescale and the length scale of the water film on the peristome surface is shown in *SI Appendix*, Fig. S3. The transport velocity, *v*, can be deduced from Stokes equation (35) as Eq. **3**:

$$v\propto \frac{e^{2}\gamma }{\mu l}\left(\frac{1}{r_{m}}-\frac{\cos\theta }{\alpha x}\right),$$ [3]

where *e* is the liquid film thickness, *μ* is the liquid dynamic viscosity, and *l* is the length of the peristome from the outer side to the inner side. According to Eq. **3**, water transport on the dry peristome surface with a contact angle of 30° scales as 0.4–0.7 mm s^−1^ which is in accordance with the experimental results (Fig. 2 *B* and *G*). The *r*_m_ and *x* of gathered water in the concavity reduces as the dry peristome becomes wet, as the data shown in Fig. 2*D* and the red curve shown in Fig. 2*F*. A much higher water transport velocity, several millimeters per second in calculation, is thus achieved on the wet peristome surface.

We statistically analyze the spreading speed, transport length, and condensation weight of 100 condensate droplets at 10 positions around the whole circle of the peristome and find the maximum spreading speeds, ranging from several millimeters per second on the wet ratchet teeth and concavities to hundreds of millimeters per second on wetted arch channels, as revealed by the blue distributions in Fig. 2*G*, respectively. Significantly, the average transport speed for the whole process increases by 300 times as the peristome surface switches from the dry state to the wet state, as the red arrow indicates in Fig. 2*G*.

Typically, water achieves an ultrafast motion speed of centimeters per seconds when drop rolls on a super–water-repellent surface or superhydrophobic surface (36), particularly because the entrapped air layer inside superhydrophobic structures can reduce the liquid solid friction. Comparing with such a fast speed without hysteresis, the condensate water can transport on the superhydrophilic peristome surface at a similar or even higher speed. The synergistic effect among ratchet, concavity, and arch structures works together to accelerate the water transport speed and achieve efficiently transporting water. The fog-harvesting ability of the peristome facilitates the formation of water on the surface to slippery interface after the drying (*SI Appendix*, Fig. S4). Pitcher plants harness this synergistic effect to effectively construct slippery surface and survive in the harsh environment. Inspired by this water-harvest strategy, we can innovate the construction of water and organic vapor harvestor and expand the scope of application.

### Artificial Multicurvature Water Harvestor.

**Fig. 3.**
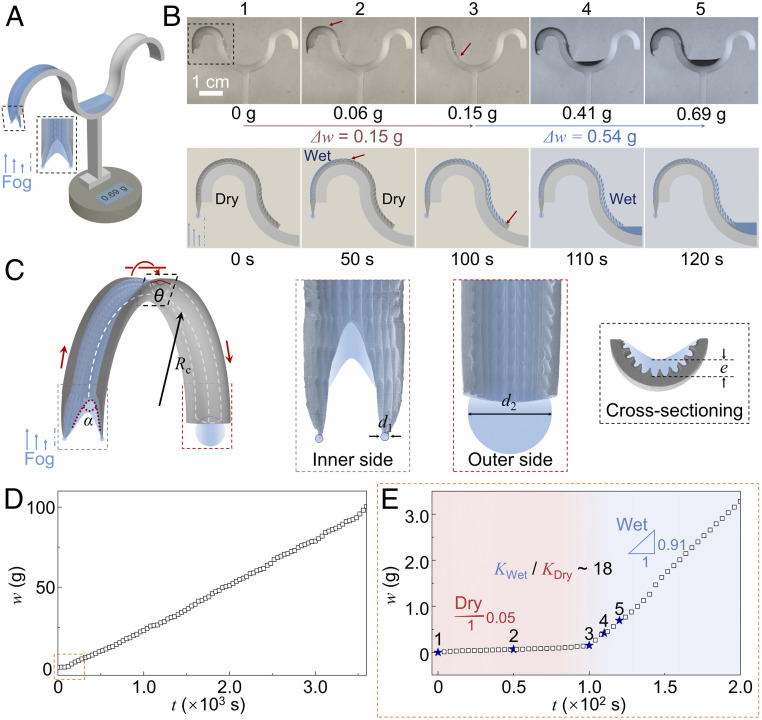
Artificial peristome water harvestor. (*A*) Schematic diagram of the artificial peristome harvestor. (*B*) Time-sequence images of the water condensation and transport process on the artificial peristome harvestor. (*C*) The mechanism for enhancing water transport speed. The water droplet with a diameter *d*_1_ condensate at the cone side transports along the wet surface to the container side. The energy release induced by the surface energy of the droplets converts to the kinetic energy for water to overflow the arch. (*D*) Water-harvest weight (*w*) versus time (*t*) for the water fog collected on the artificial peristome harvestor. (*E*) The water-harvest weight (*w*) versus time (*t*) during the initial water condensation state.

After understanding the water harvesting and transport mechanism on the natural peristome, we use a three-dimensional (3D) printing method (25, 26) to construct an artificial peristome harvestor and use PVA as the replica. The surface morphology of the artificial multicurvature water harvestor is shown in *SI Appendix*, Fig. S5. As Fig. 3*A* reveals, the artificial peristome with a single arch channel is coated onto a cup with the cone structures facing downward. The water fog is set at an upward velocity of 1.0 g s^−1^. The rising fog deposited onto the ratchet teeth overflows the arch-shaped channel and is transferred to the container at the other end. It takes 100 s to turn the artificial peristome surface from the dry state to the wet state (Fig. 3*B*). In the dry state, the average water collection speed per arch is 3.5 × 10^−3^ g cm^−2^⋅s^−1^, and the water transport speed is 4.6 mm s^−1^. After 100 s, the collected water that covers the curved surface acts as communicating vessels that connect the collecting point and the gathering point (*SI Appendix*, Fig. S6). The water channel speeds up the water-harvesting efficiency, where a water amount of 5.4 × 10^−1^ g is gathered through a wet channel during the same period of 20 s (Fig. 3*B*). There is an average water collection of 6.8 × 10^−2^ g cm^−2^⋅s^−1^, almost 20 times faster than that on the dry surface. The condensate water can slide on the water-covered surface in a high speed. The maximum water transport speed on the wet surface reaches 1.2 × 10^3^ mm s^−1^ which is about 260 times larger than that on the dry surface.

To demonstrate the role of multiscaled curvatures, peristome-mimetic half-tubes without ratchet teeth and smooth half-tubes are fabricated for comparison. As *SI Appendix*, Fig. S7 reveals, water drips down from the bare half-tube at ∼3.6 s and drips down from the peristome tube without ratchet teeth or concavity with a harvest volume of 1.9 × 10^−1^ g at ∼4.7 s. No water drips from the peristome tube with ratchet teeth. In addition, if condensate water cannot form on the surface on time, a delay in water transport occurs at the precursor side on the dry surface (19), which slows the condensate efficiency and forces condensate water to drip down. For the peristome tube with ratchet, a water-harvest volume of 2.3 × 10^−1^ g is achieved within the shortest time of 3.6 s (1.6 × 10^−1^ g cm^−2^⋅s^−1^). As a result, introducing the conical ratchet or concavity structures at the bottom edge of the peristome tube, we can increase both the water-harvesting amount and the water-harvesting speed. The artificial peristome harvestor with an opening angle of 45° for the concavity, a radius of curvature of 1.5 cm for the arch channel, and a bending angle of 180° for the channel shows the best water-harvest ability (*SI Appendix*, Figs. S8–S10). The synergistic effect of feature structures, ratchet teeth, concavities, and peristome structures plays a vital role in water harvesting. Although the wind can influence the harvest efficiency (*SI Appendix*, Figs. S11–S13), the peristome surface is a unique surface model that integrates several advantages to achieve high efficiency.

The acceleration of water transport speed is not only induced by the Laplace pressure imbalance (37) at the concavity but accelerated by the energy release, ∆*E*, of the droplets at the cone side (Fig. 3*C*). Comparing with the water motion process driven by the Laplace pressure at the concavity, the water velocity achieved by the release of surface tension of condensate droplet is 10 times higher. As Fig. 3*C* reveals, the release of surface energy at the cone side, ∆*E* ∼ *γd*_1_^2^, converts into the kinetic energy of the liquid. Considering the drop size of *d*_2_ is much larger than *d*_1_, the transport velocity, *v*, of the condensate water can be deduced as Eq. 4:

$$v\propto \sqrt{\frac{3\gamma }{\rho d_{1}}}.$$ [4]

Droplets with an average diameter *d*_1_ of 20 μm can trigger the condensate water to move from the inner side to the outer side with a typical velocity of ∼1.0 m s^−1^. The harvest speed for the water fog increases as the artificial fog harvestor turns into the wet state (Fig. 3*D*). The harvest area will increase if we pattern the single arch channel into an array. Therefore, we can further accelerate the liquid harvest amount.

### Multifunctional Liquid Harvestor.

**Fig. 4.**
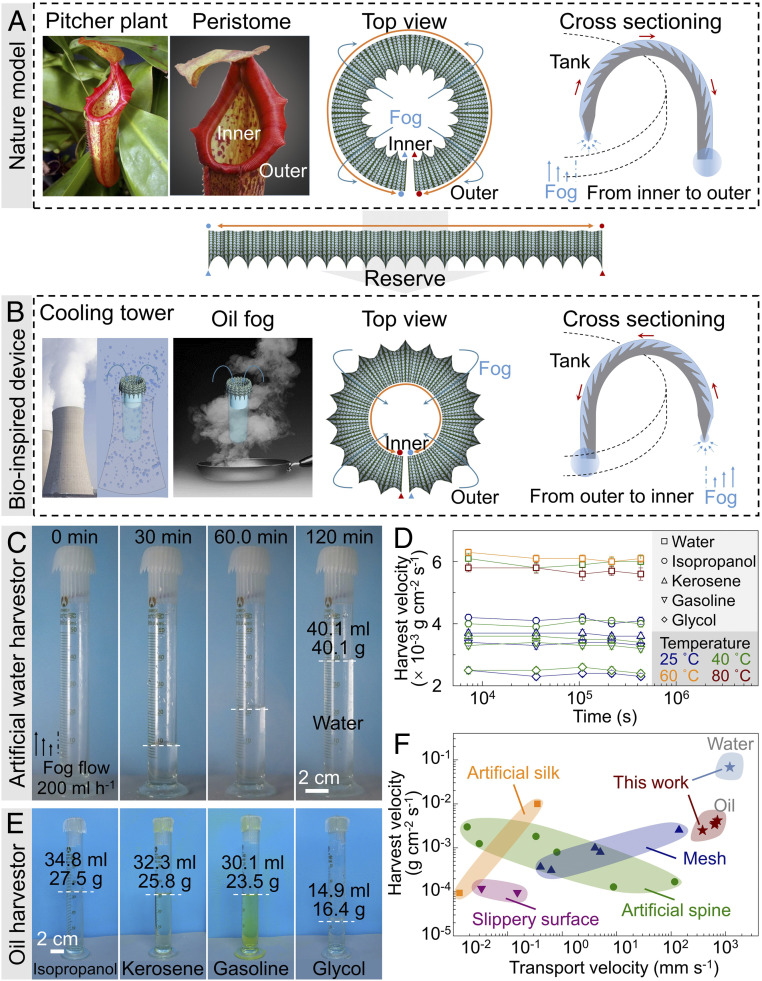
Multifunctional liquid harvestor device. (*A*) The natural peristome has a corolla shape, with the ratchet teeth facing downward on the inner side. Condensate water can be transported from the inner to the outer side, forming a slippery surface. (*B*) Reversal of the corolla, with the ratchet teeth facing downward on the outer side, could benefit the transport of water condensation from the atmosphere into the inner tank. (*C*–*E*) Both water and oil fogs are gathered and stored in the artificial peristome harvestor. (*C*) The performance of an artificial hydrogel harvestor when gathering water fog at a flow volume rate of 200 mL h^−1^. (*D*) The long-term usage of artificial water harvestor and artificial oleogel harvestor. The water-harvestor gathers can collect water fog at high temperatures. (*E*) The artificial oleogel harvestor gathers the isopropanol, kerosene, gasoline, and glycol fogs. (*F*) Transport and harvest velocity of liquid condensation on different artificial structures. See *SI Appendix*, Fig. S14 and Table S3 for details.

Natural pitcher plants can gather water fog from a pitcher tank with a circular shape (13, 22). The multicurvatures facilitate water condensation and transport from the inner side to the outer side to form a stable slippery surface (Fig. 4*A*). For practical usage, we reverse the collar direction, with the ratchet teeth facing downward on the outer side, where liquid fog can be transported from the outer side to the inner side to fill the tank (Fig. 4*B*). The biomimetic system would benefit water collection in a cooling tower and the collection of oil or organic fog (37–41) in a chemical plant, laboratory, or kitchen. In the proof-of-concept experiment, digital light procession 3D printing was used to construct peristome-mimetic substrate with invert structures. The PVA hydrogel and the PDMS oleogel replica are fabricated for the artificial water harvestor and oil harvestor by replicating the surface morphology of 3D printed substrate.

Mounting the artificial peristome along the circle of glass cylinder achieves the fog harvestor. The harvesting device is stored in a sealed glass box. A commercial Venturi atomizer is used to produce liquid fog. The fog flow is set at an upward velocity of 5.6 × 10^−2^ g s^−1^ (200 mL h^−1^). Considering the perimeter of artificial peristome circling the glass cylinder is 90 mm, the artificial peristome harvestor can gather 40.1 mL water within 2 h at an average velocity of ∼6.1 × 10^−3^ g cm^−2^⋅s^−1^ for water fog at room temperature (Fig. 4*C*). In addition, the artificial water harvestor can collect water fog at high temperature ranging from 40 to 80 °C without reducing the harvesting efficiency (Fig. 4*D*).

Comparing with water-harvesting devices, the multifunctional PDMS oleogel harvestor can harvest organic vapor at a high speed (Fig. 4 *D* and *E*). The artificial liquid harvestor can harvest organic vapor at a rate of ∼4.2 × 10^−3^ g cm^−2^⋅s^−1^ for isopropanol, ∼3.7 × 10^−3^ g cm^−2⋅^s^−1^ for kerosene, ∼3.4 × 10^−3^ g cm^−2⋅^s^−1^ for gasoline, and ∼2.5 × 10^−3^ g cm^−2⋅^s^−1^ for glycol (Fig. 4*E*, and see *SI Appendix*, Table S2 for physical and chemical properties of the liquids). These values were relatively consistent during the long-term experiments, ∼120 h (Fig. 4*D*). Traditional fog harvestor can only collect water fog. Our proposed biomimetic approach is beneficial to the construction of water and organic vapor harvestor. Significantly, our method achieves the much higher harvesting speed and transport speed than artificial harvestor inspired by spider silk, spine, and slippery pitcher surfaces (Fig. 4*F*).

## Conclusion

In summary, in view of the environmental importance of water collecting and oil fogs harvesting, we have demonstrated the water-harvest mechanism of the peristome of the *Nepenthes* plant and harnessed multicurvatures-based biomimetic structures to harvest water and oil fogs. In this work, we have introduced several key elements including: 1) The unique water collection system is composed of ratchet teeth, concavities, and arch channels on the peristome surface; each contains integrated curvatures that play different roles in the fog harvesting and transport process. 2) The surface-gradient–induced Laplace pressure at the ratchet and concavity endows the peristome with an efficient water condensation and transportation system. 3) The formation of the condensate water layer on the surface can enhance the subsequent water harvesting with a recording speed. Investigations of the structure–function relationship in this system may help us in designing novel materials and devices for collecting water from fog and in transporting condensate water with high efficiencies. We envision the multicurvature design could be customized to the shape of the specific collection devices by the 3D printing process to provide the required harvesting efficiency during the individual usage.

## Materials and Methods

### Preparation of Peristome Surfaces.

The pitcher plant investigated in this work was purchased from the XCCT Corporation. All peristome surfaces were cleaned with Milli-Q deionized water and ethanol and dried with N_2_ gas in sequence.

### Fabrication of Peristome-Mimetic Substrates.

The morphology of the 3D printed substrate was reconstructed from micro-CT scanning and redesigned by computer-aided design. The 3D printing substrate was printed by a commercial Digital light procession printer layer by layer every 30 μm with a 405-nm irritation at 61 mW by Octave Light R1 (42). After 3D printing, the printed substrate was immersed in ethanol for 5 min to remove the uncured resin. Artificial hydrophilic PVA hydrogel replica was fabricated by replicating the surface morphology of 3D printed substrate. The PVA powder was dispersed in a liquid mixed by water and dimethyl sulfoxide (DMSO) at room temperature for half an hour to swell. The mass proportion of PVA:water:DMSO is 20:45:135. The dispersion liquid was then heated in water bath at a temperature of 90 °C and stirred at 400 rpm for 4 h to fully dissolve the PVA powder. Pouring the PVA solution onto the printed substrate and storing in a refrigerator at −20 °C for 6 h, PVA hydrogel replica was prepared. By soaking the preprocessed replica in ethanol for 2 h, the replica shrunk in size and had a 10 times higher resolution. The PDMS oleogel replica was achieved by copying the morphology of the 3D printed model by mixing the Dow silicone 10–1 encapsulants. When the two-part liquid component kits are thoroughly mixed and cured at 80 °C for 4 h, the mixture cures to a flexible elastomer with the peristome-mimetic morphology.

### Characterization.

The water harvest on each real/artificial peristome surface was recorded with a FASTCAM MX100 high-speed camera (Photron) and digital camera, Nikon D7500, at relative humidity of ∼95%. The fog was controlled by a humidifier. The weight of gathered liquid is recorded with a microbalance (ME204, Mettler) in real time. ZEISS Discovery V8 stereomicroscope was equipped with a high-speed camera, FASTCAM MX100, to record water condensation at the ratchet tips.

SEM images were obtained using a field-emission SEM (Hitachi-S8010, Phenom Pro X). The preparation steps of the peristome samples for SEM observation included three steps: fixed geometry at 4 °C for 24 h by 90 mL 50% alcohol, 5 mL glacial acetic acid and 5 mL formalin; dehydration by immersed in a series of alcohol with concentrations from 50, 70, 85, 95, and 100%. Each lasted for a minimum of 1 h and ended with 100% ethanol overnight; shoot apices of peristome were subjected to vacuum infiltration in a fixative solution (5% formaldehyde, 5% acetic acid, and 50% ethanol) for 30 min and then kept at room temperature overnight. The peristome was mounted on aluminum stubs, dissected under a stereomicroscope, and sputter-coated with gold. Peristome was analyzed by high-resolution SEM with an accelerating voltage of 5 kV.

For videoing of the water condensation on the ratchet of the peristome, peristome was transferred onto alumina supports to manage the temperature. For cross-sectional studies, the peristome was prepared by soaking in ethanol and then freeze-fractured in liquid nitrogen and dried in dry air. The condensation video was obtained using the Environmental SEM, QUANTA FEG 250.

High-resolution 3D X-ray microscopy and computed tomography images were obtained with a SKYSCAN 1272 from Bruker. Individual X-ray exposure slices were used to construct 3D copies of the samples.

To study the surface wetting behavior, water contact angle measurements were performed with a contact angle measurement device, LSA 100 Surface Analyzer (LAUDA Scientific) at room temperature. All samples were dried at room temperature prior to measuring their contact angles. A drop of water, 3 µl, was placed on the sample surface using a micropipette. The contact angle was measured using a circle fitting method by drop-shape analysis software. Each reported contact angle was an average of at least five independent measurements. Average value of contact angles are presented with the SD.

## Supplementary Material

Supplementary File

Supplementary File

Supplementary File

Supplementary File

Supplementary File

Supplementary File

Supplementary File

Supplementary File

Supplementary File

Supplementary File

## Data Availability

All study data are included in the article and *SI Appendix*.
