# Supplementary material for: Liquid harvesting and transport on multiscaled curvatures
Source: Proc Natl Acad Sci U S A. 2020 Sep 8;117(38):23436–42. doi: 10.1073/pnas.2011935117 (PMC7519342; doi:10.1073/pnas.2011935117)
Supplement: Supplementary File [file pnas.2011935117.sapp.pdf]

PNAS
www.pnas.org

Supplementary Information for

Liquid harvesting and transport on multi-scaled curvatures

Chuxin Li, Cunlong Yu, Shan Zhou, Zhichao Dong*, and Lei Jiang

* Zhichao Dong
Email: dongzhichao@iccas.ac.cn

This PDF file includes:

Supplementary text
Figures S1 to S14
Tables S1 to S3
Legend for Movie S1 and Movie S2
Legend for Dataset S1
SI References

Other supplementary materials for this manuscript include the following:

Movie S1
Movie S2
Dataset S1-S7

www.pnas.org/cgi/doi/10.1073/pnas.2011935117

Supplementary Information Text

Threshold dripping size on tilted surface. Traditionally, the collection of the sessile fog drops is driven by the gravity-drainage (1). The shedding of a sessile drop with radius R on the inclined surface with tilt angle α starts when the gravitational force ~ $\rho g \sin\alpha R^3$, where ρ is the liquid density, and g is the gravity, overcomes the pinning force γ ($\cos\theta_r - \cos\theta_a$) R induced by the wetting hysteresis, where γ is the surface tension, and θ_a and θ_r are the advancing and receding contact angles, respectively.

Sliding a gathered drop (2, 3) on a titled substrate requires the threshold drop radius to be above a large size. Balancing the gravitational force and the pinning force, the threshold sliding size can be regarded as:

$$R = \sqrt{\frac{(\cos\theta_{\mathrm{r}} - \cos\theta_{\mathrm{a}})\,\gamma}{\rho g \sin\alpha}} \qquad \text{[S1]}$$

Considering the capillary length l_c equals $(\gamma / \rho g)^{1/2}$, the critical depinning drop radius follows:

$$R = l_{\mathrm{c}}\sqrt{\frac{(\cos\theta_{\mathrm{r}} - \cos\theta_{\mathrm{a}})}{\sin\alpha}} \qquad \text{[S2]}$$

Fog collection is therefore limited by the transient stage before the onset of shedding.

Threshold tilt angle. The shedding of a sessile drop with a smaller radius need to increase the tilt angle. Balancing the gravitational force and the pinning force, the threshold tilt angle can be regarded as:

$$\alpha = \sin^{-1} d\,\frac{(\cos\theta_{\mathrm{r}} - \cos\theta_{\mathrm{a}}){l_{\mathrm{c}}}^2}{R^2} \qquad \text{[S3]}$$

Therefore, the larger the tilt angle, the smaller the depinning drop radius.

Water harvest on peristome surface. In strong contrast to pervious water harvestor, the peristome has concave structures in between ratchets. The concave of the peristome surface can hold the condensation water without dripping down. The water inside the concave with a height h is supported by the Laplace pressure difference between the two menisci. Considering water menisci pins at a distance of x from the centre with the menisci radius, r_m, in the cross sectioning (Fig. S2), the Laplace pressure is regarded as:

$$\Delta P = \gamma\left(\frac{1}{r_{\mathrm{m}}} - \frac{\cos\theta}{\alpha x}\right) \qquad \text{[S4]}$$

where γ is the water surface tension, θ is the water contact angle, and α is the opening angle of the concavity, respectively. Balancing the hydrostatic pressure, ρgH, and the Laplace pressure, ΔP, we can achieve the threshold water climbing height as:

$$H_{\max} = {l_{\mathrm{c}}}^2\left(\frac{1}{r_{\mathrm{m}}} - \frac{\cos\theta}{\alpha x}\right) \qquad \text{[S5]}$$

Driving by the Laplace pressure, the condensate water can transport on the arch and overflow the peristome from the inner side to the outer side.

Beside the water collection ability, the concave can accelerate the transport speed of the condensate water. In the common case of an incompressible Newtonian fluid, the Navier-Stokes equations take the form:

$$\rho\frac{\partial \mathbf{u}}{\partial t} + \rho(\mathbf{u}.\nabla)\mathbf{u} = -\nabla p + \eta\Delta\mathbf{u} + \rho\mathbf{f} \qquad \text{[S6]}$$

where the inertia part $\rho\frac{\partial \mathbf{u}}{\partial t} + \rho(\mathbf{u}.\nabla)\mathbf{u}$ is ~ $\rho u^2/l$, the pressure gradients part $-\nabla p$ is $\Delta P/l$, the viscosity part $\eta\Delta\mathbf{u}$ is ~ $\eta\mathbf{u}/e^2$, and the body forces is $\rho\mathbf{f}$ with $\mathbf{u}$ the velocity of the fluid, ∇p the gradient of the pressure and μ the dynamic viscosity (4). The film flows along the peristome as a result of the pressure difference between the two menisci. Balancing the inertia and viscosity part, we achieve:

$$\mu\Delta\mathbf{u} - \boldsymbol{\nabla} p = \mathbf{0} \qquad \text{[S7]}$$

$$\frac{\Delta P}{l} = \frac{\mu v}{e^2} \quad \text{[S8]}$$

and the film transport velocity v scales as:

$$v \propto \frac{e^2 \gamma}{\mu l}\left(\frac{1}{r_{\mathrm{m}}} - \frac{\cos\theta}{\alpha x}\right) \quad \text{[S9]}$$

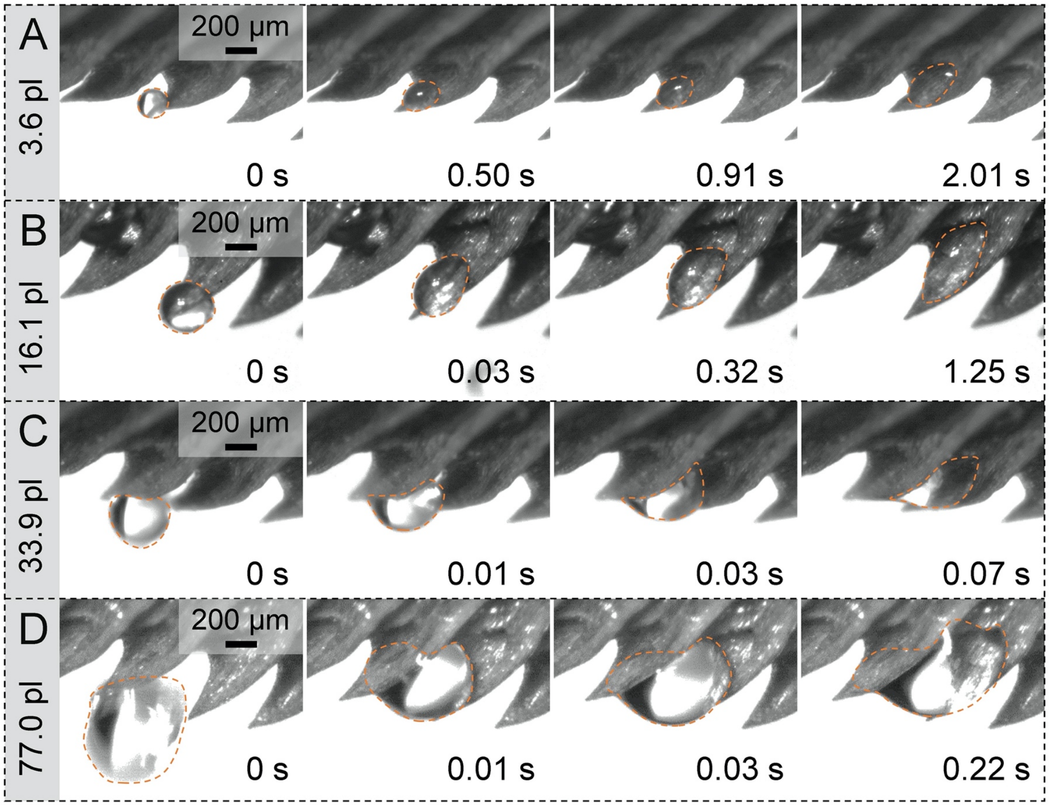

Fig. S1. Water droplets captured by ratchet teeth. (*A*-*D*) Selected snapshots of water droplets, ranging from 3.6 pl to 77.0 pl, impinging and directional transport on the teeth of peristome. During the drop impacting process, the ratchet and concavity can generate driving force for the directional transport.

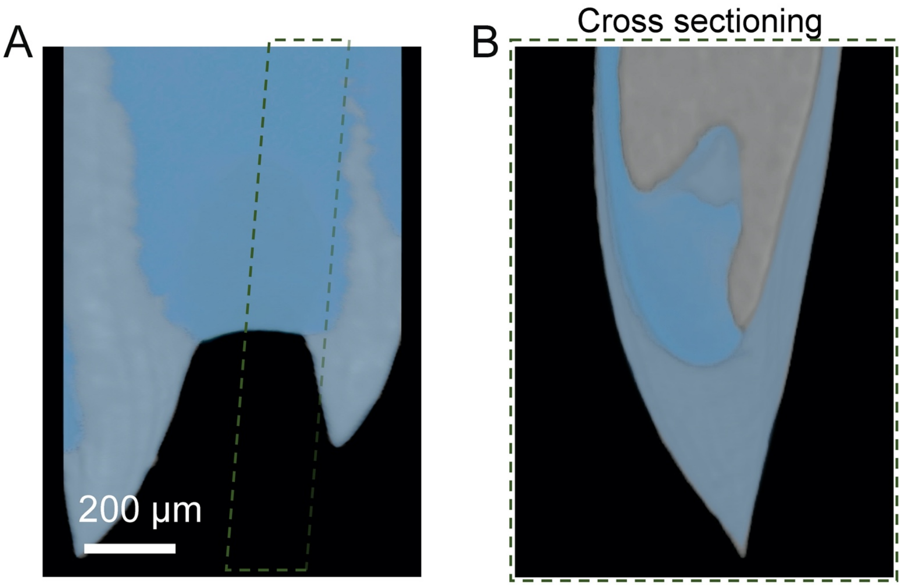

Fig. S2. Micro-CT images of water inside the concavity. (*A*) The water inside concavity has a concave meniscus from the front view. (*B*) The meniscus is convex from the cross sectioning view.

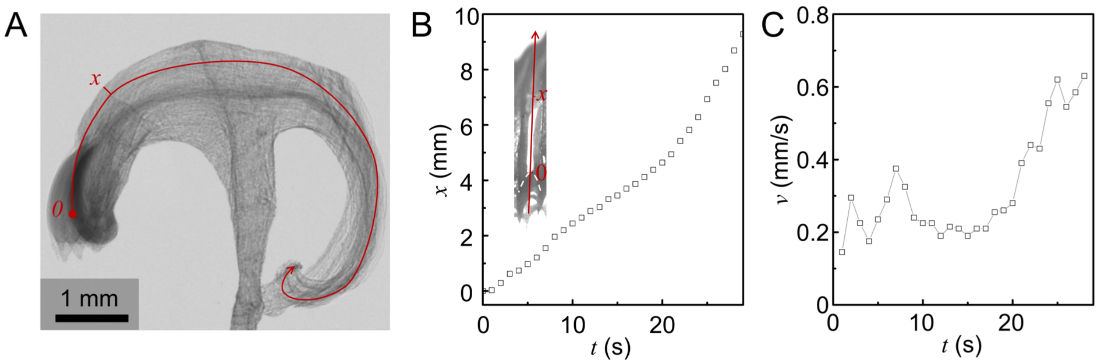

Fig. S3. Correlation between the time scale and length scale of the water film formed on the peristome surface. (*A*) X-ray image of the *Nepenthes alata* pitcher indicating a thin water film covers the arch-shaped peristome surface. (*B*) Water transport distance (*x*) from the concavity tip as a function of time (*t*). (*C*) Water transport velocity (*v*) of the front of the precursor as a function of time (*t*).

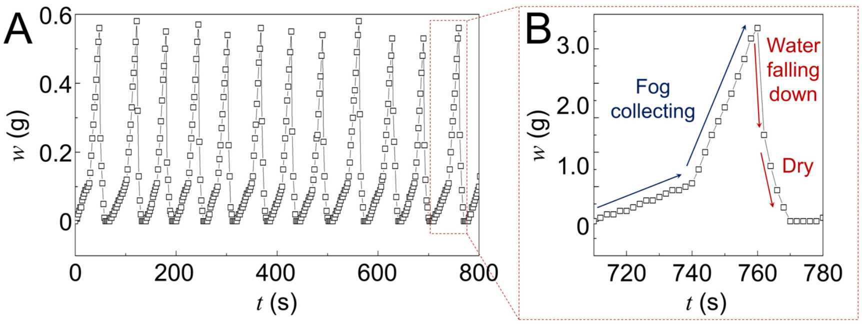

Fig. S4. Fog harvesting behavior on natural peristome surface for 800 s. (*A*) Liquid harvest weight (*w*) versus time (*t*) for the water fog collected on the artificial peristome harvestor. (*B*) Enlarged diagram within the time ranged from 710 s to 780 s.

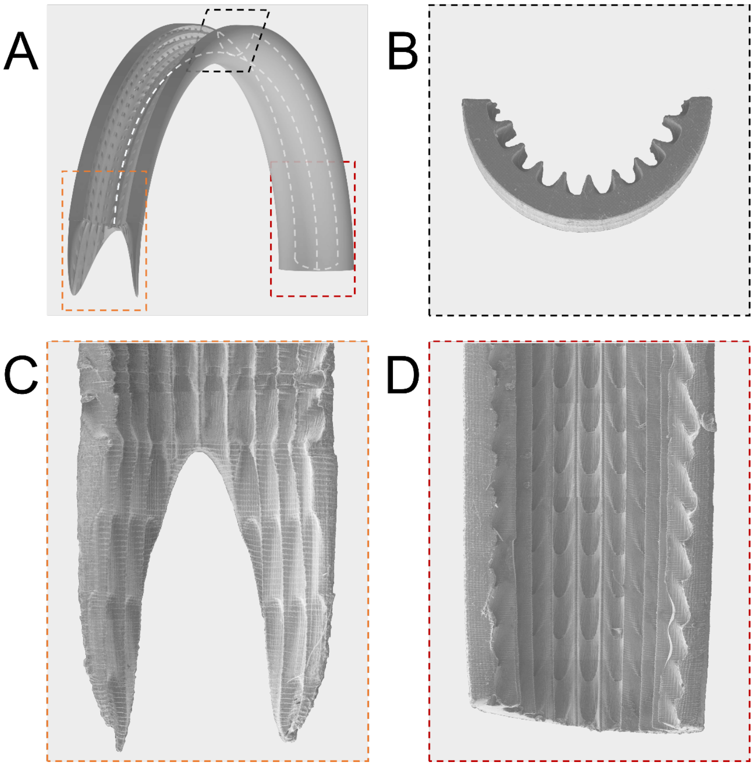

Fig. S5. Schematic and SEM images of the artificial peristome harvestor. (*A*) Schematic image of the artificial peristome harvestor. (*B-D*) The feature structures of the artificial peristome harvestor. (*B*) Cross sectioning SEM image of the channel. (*C*) SEM image of the ratchet side of the channel. (*D*) SEM image of the peristome surface at the other side of the channel.

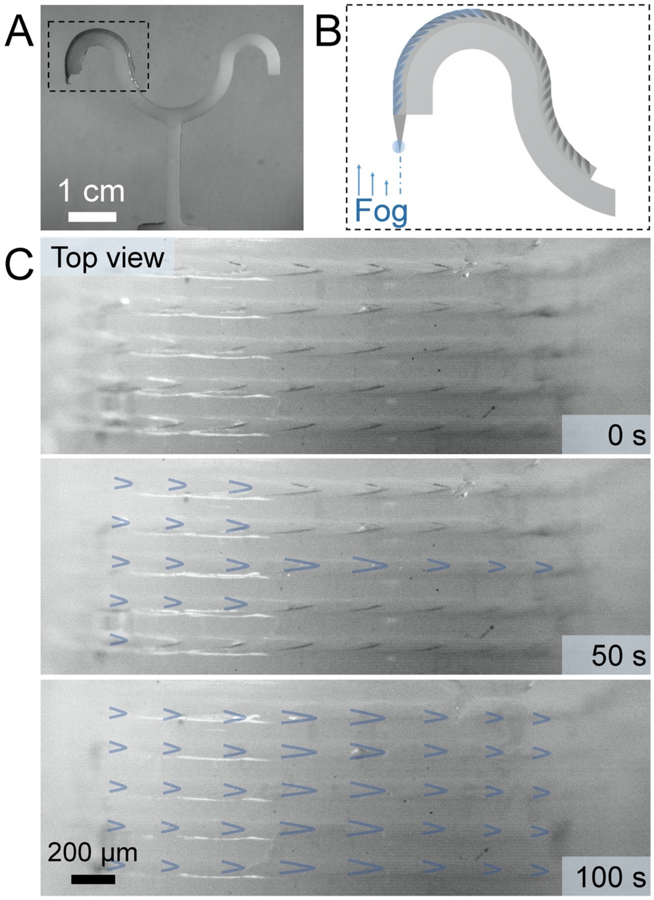

Fig. S6. Fogs harvest and transport behaviors on the artificial peristome harvestor. (*A*) The optical image of the artificial peristome harvestor. (*B*) Schematic diagram of the artificial peristome harvestor. (*C*) Condensate liquid overflows the arch-shaped channel and covers the micro-scaled curvatures.

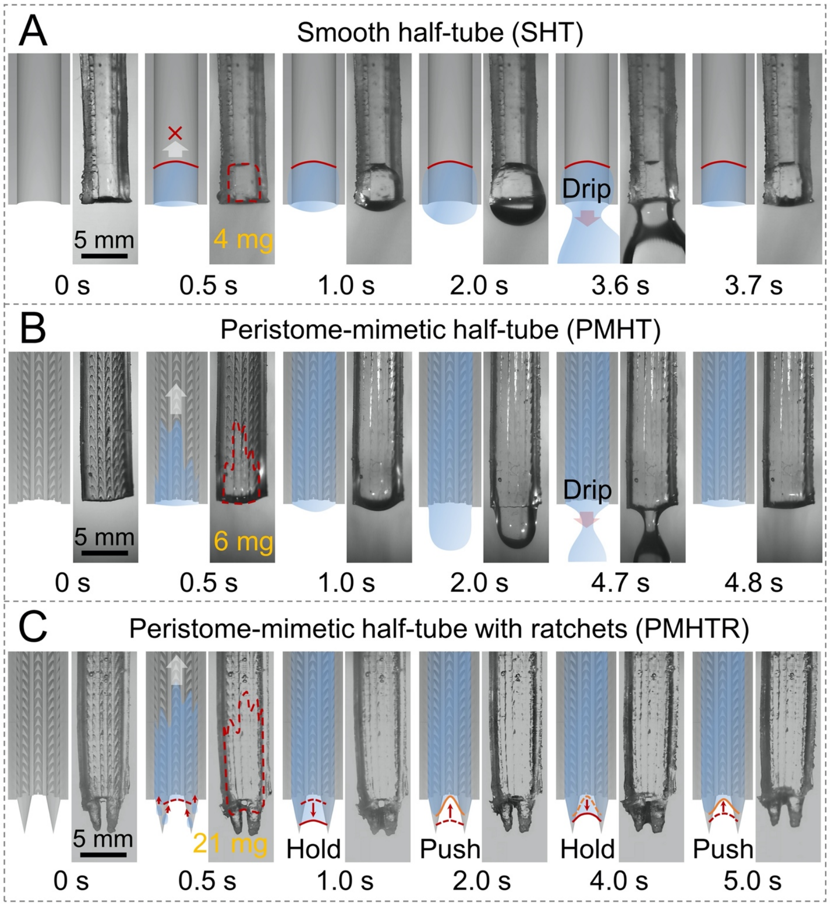

Fig. S7. Water condensation and transport on the artificial substrates. (*A*) Water condensation on the smooth PVA half-tube with a limited water elevating height. (*B*) Water condensation on the peristome-mimetic half-tube. The collected water can transport along the pre-wetted peristome-mimetic half-tube. (*C*) The additional ratchets and concavity on the peristome-mimetic half-tube largely enhance the fog harvesting amount as well as the harvest speed.

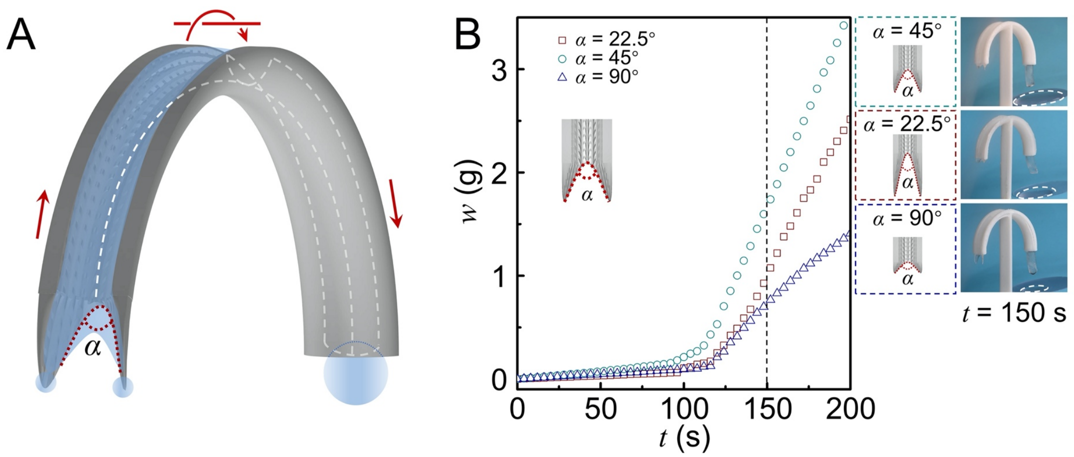

Fig. S8. Water condensation and transport on artificial peristome harvester with different opening angles at the concavity. (*A*) Schematic diagram of the artificial peristome harvester. Water condensation on the peristome-mimetic surface with different opening angles, *α*, ranging from 22.5º to 90º. (*B*) Liquid harvest weight (*w*) versus time (*t*). The artificial peristome harvester with a bending angle of 45º shows the best water harvest ability.

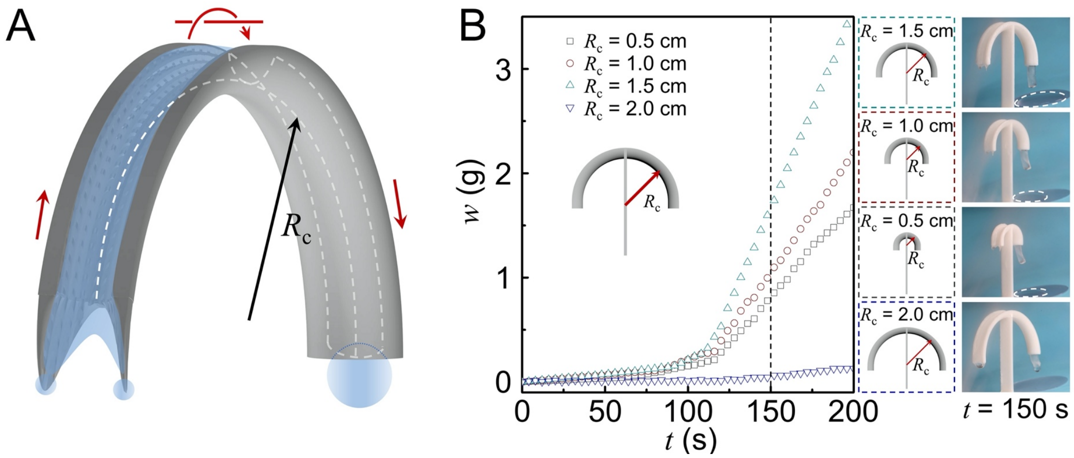

Fig. S9. Water condensation and transport on artificial peristome harvestor with different radii of curvatures. (*A*) Schematic diagram of the artificial peristome harvestor. Water condensation on the artificial peristome harvestor with different radii of curvatures, ranging from 0.5 cm to 2.0 cm. (*B*) Liquid harvest weight (*w*) versus time (*t*). The artificial water collector with a radius of curvature of 1.5 cm shows the best water harvest ability.

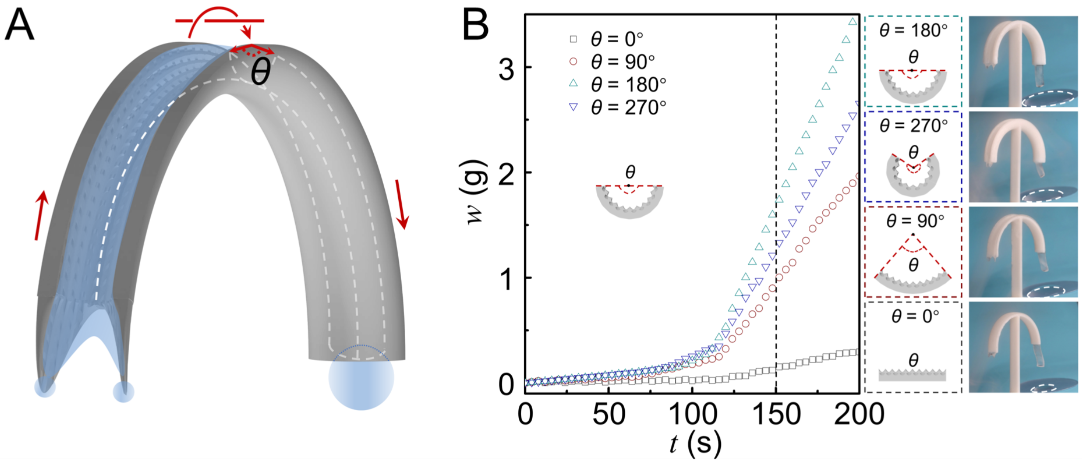

Fig. S10. Water condensation and transport on artificial peristome harvestor with different bending angles at the channel. (*A*) Schematic diagram of the artificial peristome harvestor. Water condensation on the artificial peristome harvestor with different bending angles of the channel, ranging from 0° to 270°. (*B*) Liquid harvest weight (*w*) versus time (*t*). The artificial water collector with bending angle of 180° at the channel shows the best water harvest ability.

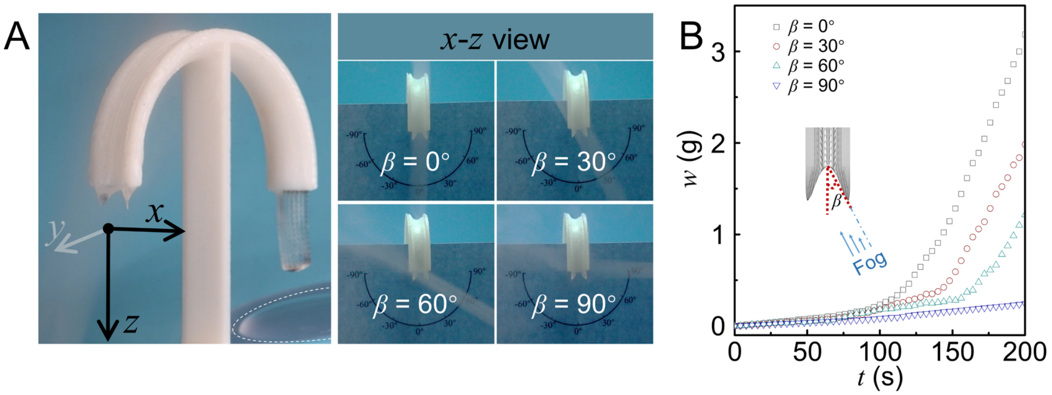

Fig. S11. Water condensation and transport on artificial peristome harvestor with different wind direction from the *x*-*z* view. (*A*) Optical image of the artificial peristome harvestor. Water condensation on the peristome-mimetic surface with different wind direction, *β*, ranging from 0º to 90º from the *x*-*z* view. (*B*) Liquid harvest weight (*w*) versus time (*t*). The artificial peristome harvestor with wind direction of 0º from *x*-*z* view shows the best water harvest ability.

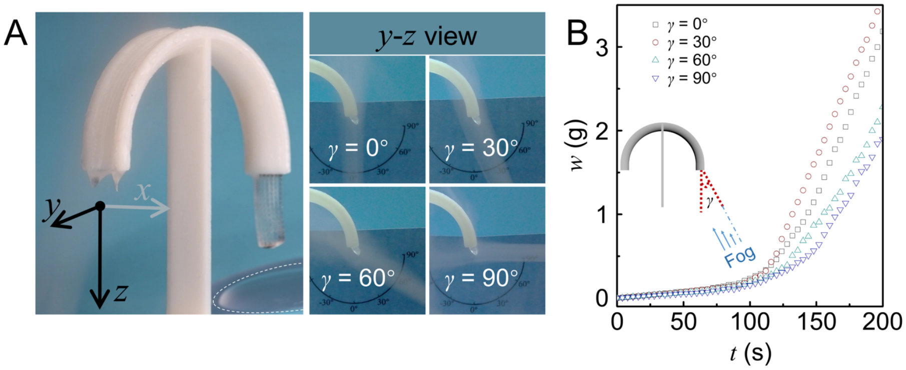

Fig. S12. Water condensation and transport on artificial peristome harvestor with different wind direction from the *x*-*z* view. (*A*) Optical image of the artificial peristome harvestor. Water condensation on the peristome-mimetic surface with different wind direction, *β*, ranging from 0º to 90º from the *x*-*z* view. (*B*) Liquid harvest weight (*w*) versus time (*t*). The artificial peristome harvestor with wind direction of 0º from *x*-*z* view shows the best water harvest ability.

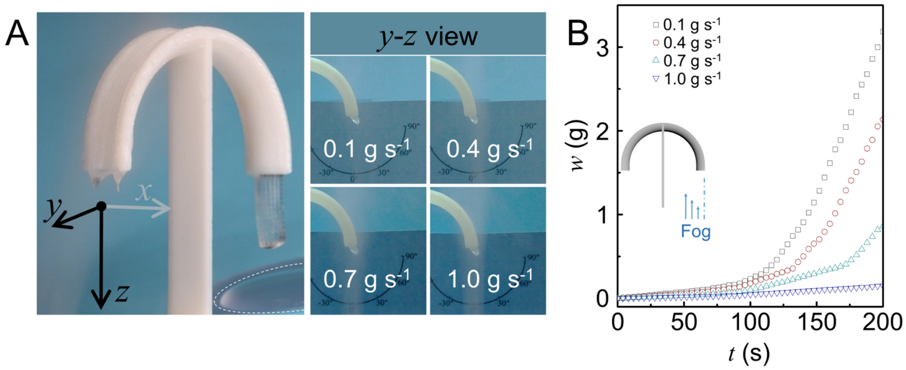

Fig. S13. Water condensation and transport on artificial peristome harvestor with different water fog velocity. (*A*) Optical image of the artificial peristome harvestor. Water condensation on the peristome-mimetic surface with different water fog velocity, ranging from 0.1-1.0 g s^{-1}. (*B*) Liquid harvest weight (*w*) versus time (*t*). The artificial peristome harvestor with water fog velocity of 1.0 g s^{-1} shows the best water harvest ability.

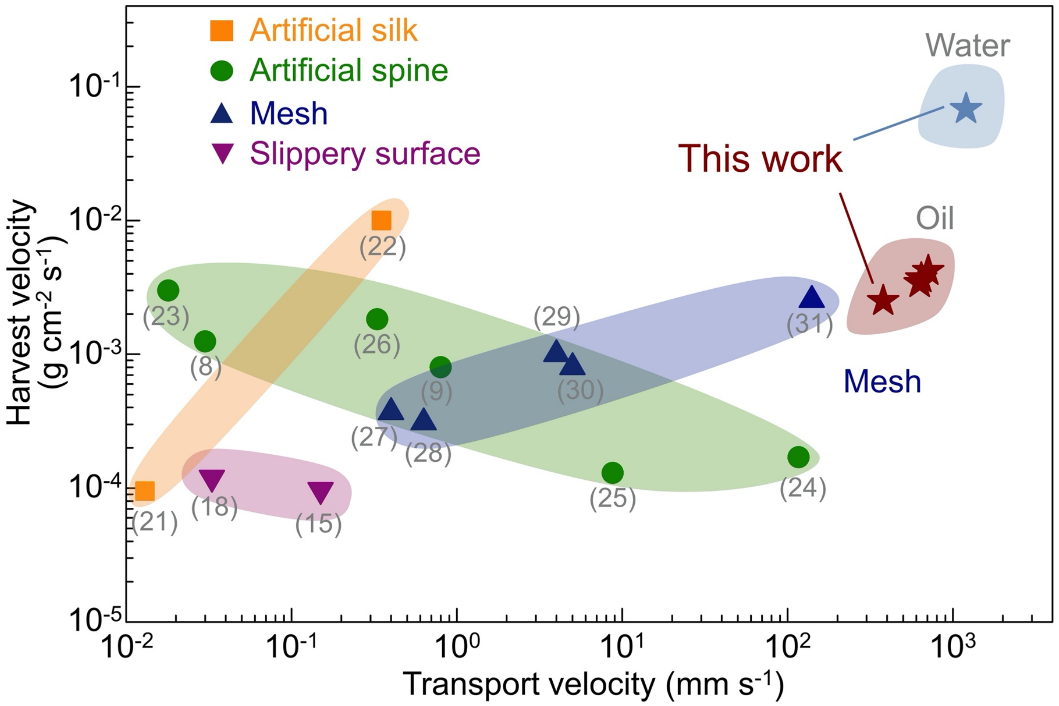

Fig. S14. Transport and harvest velocity of liquid condensation on different natural and artificial structures.

Table S1. Experimental liquid transport velocities on natural or artificial surfaces.

Substrate	Study	Liquid	Transport velocity (μm/s)	Ref.
Cactus spine	Ju *et al.* (**Nat. Commun.**, 2012)	Water	~ 13	(5)
Artificial spine	Lorenceau *et al.* (**J. Fluid Mech.**, 2004)	Silicone oil	~ 6,000	(6)
	Li *et al.* (**Nat. Commun.**, 2013)	Silicone oil	~ 2,000	(7)
	Cao *et al.* (**Adv. Funct. Mater.**, 2014)	Water	~ 30	(8)
	Zhang *et al.* (**Proc. Natl. Acad. Sci. U.S.A.**, 2019)	Water	~ 150	(9)
	Jiang *et al.* (**Proc. Natl. Acad. Sci. U.S.A.**, 2019)	Water	~ 10,000	(10)
Spider silk	Zheng *et al.* (**Nature**, 2010)	Water	~ 35	(11)
Artificial silk	Bai *et al.* (**Adv. Mater.**, 2010)	water	~ 1,000	(12)
	Bai *et al.* (**Adv. Mater.**, 2011)	Water	~21	(13)
Artificial structures	Lv *et al.* (**Phys. Rev. Let.**, 2014)	Water	~ 180	(14)
	Park *et al.* (**Nature**, 2016)	Water	~ 150	(15)
Syntrichia caninervis	Pan *et al.* (**Nat. Plants**, 2016)	Water	~90	(16)
Sarracenia trichome	Chen *et al.* (**Nat. Mater.**, 2018)	Water	~ 11,700	(17)
Pitcher plant	Chen *et al.* (**Nature**, 2016)	Water	~ 8,000	(18)
	Li *et al.* (**Angew. Chem. Int. Ed.**, 2016)	Water	~ 1,000	(19)
	Li *et al.* (**Sci. Adv.**, 2017)	Water	~ 6,300	(20)
Pitcher plant	**This work**	Water	~ 187,444	

Table S2. Physicochemical properties of the liquids at 20°C.

Name	Surface tension, γ (mN m^{-1})	Viscosity, μ (mPa s)	Density, ρ (g cm^{-3})	Boiling point, T_b (°C)	Explosion limits, (%)
Water	72.0	0.89	1.00	100.0	/
Isopropanol	21.7	2.37	0.79	82.5	2.0-12.0
Kerosene	24.1	2.50	0.80	180-310	0.7-5.0
Gasoline	22.0	0.78	0.78	30-205	1.4-7.6
Glycol	47.7	19.9	1.11	189.0	3.2-15.3

Table S3. Transport and harvest velocity of liquid condensation on different natural and artificial structures.

Substrate	Study	Transport velocity (mm s^{-1})	Harvest velocity (g cm^{-2} s^{-1})	Ref.
Artificial silk	Chen *et al.* (**Soft Matter**, 2012)	~ 0.013	~ 9.50×10^{-5}	(21)
	Dong *et al.* (**Adv. Mater. Interfaces**, 2016)	~ 0.35	~ 1.00×10^{-2}	(22)
Artificial spine	Ju *et al.* (**Adv. Funct. Mater.**, 2014)	~ 0.02	~ 3.00×10^{-3}	(23)
	Cao *et al.* (**Adv. Funct. Mater.**, 2014)	~ 0.03	~ 1.30×10^{-3}	(8)
	Xu *et al.* (**ACS Nano**, 2016)	~ 116.70	~ 1.72×10^{-4}	(24)
	Lin *et al.* (**ACS Appl. Mater. Interfaces**, 2018)	~ 8.75	~ $1.3.0 \times 10^{-4}$	(25)
	Yi *et al.* (**Adv. Mater. Technol.**, 2019)	~ 0.33	~ 1.83×10^{-3}	(26)
	Zhang *et al.* (**Proc. Natl. Acad. Sci. U.S.A.**, 2019)	~ 0.80	~ 8.00×10^{-4}	(9)
Mesh	Zhu *et al.* (**Chem. Commun.**, 2016)	~ 0.40	~ 3.66×10^{-4}	(27)
	Azad *et al.* (**Langmuir.**, 2017)	~ 0.63	~ 3.10×10^{-4}	(38)
	Damak *et al.* (**Sci. Adv.**, 2018)	~ 4.00	~ 1.04×10^{-3}	(29)
	Shi *et al.* (**ACS Appl. Mater. Interfaces**, 2018)	~ 5.00	~ 8.33×10^{-4}	(30)
	Wang *et al.* (**RSC Adv.**, 2020)	~ 140.00	~ 2.54×10^{-3}	(31)
Slippery surface	Park *et al.* (**Nature**, 2016)	~ 0.15	~ 9.70×10^{-5}	(15)
	Dai *et al.* (**Sci. Adv.**, 2018)	~ 0.03	~ 1.20×10^{-4}	(18)
This work	water	~ 1,200.00	~ 6.80×10^{-2}	
	isopropanol	~ 710.00	~ 4.20×10^{-2}	
	kerosene	~ 640.00	~ 3.70×10^{-2}	
	gasoline	~ 630.00	~ 3.40×10^{-2}	
	glycol	~ 380.00	~ 2.50×10^{-2}	

Movie S1 (separate file). Zooming the surface structures of the peristome in 3D rotation view.

Movie S2 (separate file). Fog collect and transport on the peristome surface and artificial water collector.

Dataset S1-S7 (separate file). PDF file with raw data for water transport and harvest measurements. Dataset S1-S7 include data for Fig. 1H, Fig. 2D, Fig. 2G, Fig. 3D, Fig. 3E, Fig. 4D and Fig. 4F, respectively.

SI References

1. P. B. Bintein, H. Lhuissier, A. Mongruel, L. Royon, D. Beysens, Grooves accelerate dew shedding. *Phys. Rev. Lett.* 122, 098005 (2019).
2. C. Furmidge, Studies at phase interfaces. I. The sliding of liquid drops on solid surfaces and a theory for spray retention. *J. Colloid Sci.* 17, 309-324 (1962).
3. C. W. Extrand, A. N. Gent, Retention of liquid drops by solid surfaces. *J. Colloid Interface Sci.* 138, 431-442 (1990).
4. L. G. Leal, Advanced transport phenomena: fluid mechanics and convective transport processes, (Cambridge University Press, 2007), pp.686-690.
5. J. Jie, *et al*. A multi-structural and multi-functional integrated fog collection system in cactus. *Nat. Commun.* 3, 1247 (2012).
6. E. Lorenceau, D. Quéré, Drops on a conical wire. *J. Fluid Mech.* 510, 29-45 (2004).
7. K. Li, *et al*. Structured cone arrays for continuous and effective collection of micron-sized oil droplets from water. *Nat. Commun.* 4, 2276 (2013).
8. M. Cao, *et al*. Facile and large-scale fabrication of a cactus-inspired continuous fog collector. *Adv. Funct. Mater.* 24, 3235-3240 (2014).
9. X. X. Zhang, *et al*. Multibioinspired slippery surfaces with wettable bump arrays for droplets pumping. *Proc. Natl. Acad. Sci. U.S.A.* 116, 20863-20868 (2019).
10. J. K. Jiang, *et al*. Directional pumping of water and oil microdroplets on slippery surface. *Proc. Natl. Acad. Sci. U.S.A.* 116, 2482-2487 (2019).
11. Y. Zheng, *et al*. Directional water collection on wetted spider silk. *Nature* 463, 640-643 (2010).
12. H. Bai, *et al*. Direction controlled driving of tiny water drops on bioinspired artificial spider silks. *Adv. Mater.* 22, 5521-5525 (2010).
13. H. Bai, *et al*. Controlled fabrication and water collection ability of bioinspired artificial spider silks. *Adv. Mater.* 23, 3708-3711 (2011).
14. C. J. Lv, *et al*. Substrate curvature gradient drives rapid droplet motion. *Phys. Rev. Lett.* 113, 026101 (2014).
15. K.-C. Park, *et al*. Condensation on slippery asymmetric bumps. *Nature* 531, 78-82 (2016).
16. Pan *et al.* The upside-down water collection system of *Syntrichia caninervis. Nat. Plants.* 2, 16076 (2016).
17. H. Chen, *et al*. Ultrafast water harvesting and transport in hierarchical microchannels. *Nat. Mater.* 17, 935-942 (2018).
18. H. Chen, *et al*. Continuous directional water transport on the peristome surface of *Nepenthes alata*. *Nature* 532, 85-89 (2016).
19. C. Li, *et al*. Uni-directional transportation on peristome-mimetic surfaces for completely wetting liquids. *Angew. Chem. Int. Ed.* 55, 14988-14992 (2016).
20. J. Li, *et al*. Topological liquid diode. *Sci. Adv.* 3, eaao3530 (2017).
21. Y. Chen, *et al.* Bioinspired spindle-knotted fibers with a strong water-collecting ability from a humid environment. *Soft Matter* 8, 11450-11454 (2012).
22. H. Dong, *et al.* Highly efficient fog collection unit by integrating artificial spider silks. *Adv. Mater. Interfaces* 3, 1500831 (2016).
23. J. Ju, *et al.* Cactus stem inspired cone-arrayed surfaces for efficient fog collection. *Adv. Funct. Mater.* 24, 6933-6938 (2014).

24. T. Xu, *et al.* High-efficiency fog collector: water unidirectional transport on heterogeneous rough conical wires. *ACS Nano* 10, 10681-10688 (2016).
25. J. Lin, *et al.* Leaf vein-inspired hierarchical wedge-shaped tracks on flexible substrate for enhanced directional water collection. *ACS. Appl. Mater. Interfaces* 10, 44815-44824 (2018).
26. S. Yi, *et al.* Cactus-inspired conical spines with oriented microbarbs for efficient fog harvesting. *Adv. Mater. Technol.* 4, 1900727 (2019).
27. H. Zhu, *et al.* High-efficiency water collection on biomimetic material with superwettable patterns. *Chem. Commun.* 52, 12415-12417 (2016).
28. M. A. K. Azad, *et al.* Fog collection on polyethylene terephthalate (PET) fibers: influence of cross section and surface structure. *Langmuir* 33, 5555-5564 (2017).
29. M. Damak, K. K. Varanasi, Electrostatically driven fog collection using space charge injection. *Sci. Adv.* 4, eaao5323 (2018).
30. W. W. Shi, *et al.* Fog harvesting with harps. *ACS. Appl. Mater. Interfaces* 10, 11979-11986 (2018).
31. X. K. Wang, *et al.* Beetle-like droplet-jumping superamphiphobic coatings for enhancing fog collection of sheet arrays. *RSC Adv.* 10, 282-288 (2020).
