# Supplementary material for: Liquid harvesting and transport on multiscaled curvatures
Source: Proc Natl Acad Sci U S A. 2020 Sep 8;117(38):23436–42. doi: 10.1073/pnas.2011935117 (PMC7519342; doi:10.1073/pnas.2011935117)
Supplement: Supplementary File [file pnas.2011935117.sd01.pdf]

Fig. 1*H* Transport velocity of water condensation on different natural and artificial structures.

Surfaces	Transport velocity (μm s^{-1})
Natural cactus spine	~ 13
Artificial cactus spine	~ 30
Natural Spider silk	~ 35
Artificial Spider silk	~ 21
Natural *Sarracenia* trichome	11738 ± 1411
Artificial *Sarracenia* trichome	6931 ± 766
Concavity of Nepenthes peristome	7644 ± 917
Arch channel of Nepenthes peristome	187443 ± 6814
