# Supplementary material for: Liquid harvesting and transport on multiscaled curvatures
Source: Proc Natl Acad Sci U S A. 2020 Sep 8;117(38):23436–42. doi: 10.1073/pnas.2011935117 (PMC7519342; doi:10.1073/pnas.2011935117)
Supplement: Supplementary File [file pnas.2011935117.sd02.pdf]

Fig. 2*D* Plots of the water meniscus distance (x) from the concavity tip measured in (*B*) as a function of time (t).

t (s)	x (10^{-1} mm)
8.0	5.5
8.2	5.4
8.4	5.4
8.6	5.3
8.8	5.3
9.0	5.2
9.2	5.2
9.4	5.1
9.6	5.0
9.8	4.9
10.0	4.8
10.2	4.8
10.4	4.8
10.6	4.9
10.8	5.0
11.0	5.1
11.2	5.1
11.4	5.2
11.6	5.3
11.8	5.4
12.0	5.3
12.2	5.2
12.4	5.1
12.6	5.1
12.8	5.0
13.0	5.0
13.2	4.9
13.4	4.8
13.6	4.8
13.8	4.7
14.0	4.8
14.2	4.9
14.4	5.0
14.6	5.1
14.8	5.1
15.0	5.2
15.2	5.3
15.4	5.2
15.6	5.1
15.8	5.0
16.0	4.9
16.2	4.9
16.4	4.8
16.6	4.6
16.8	4.6
17.0	4.7
17.2	4.8
17.4	4.9
17.6	4.9
17.8	5.0
18.0	5.1
18.2	5.3
18.4	5.1
18.6	5.0
18.8	4.9
19.0	4.9
19.2	4.8
19.4	4.7
19.6	4.5
19.8	4.5
20.0	4.4
20.2	4.5
20.4	4.5
20.6	4.6
20.8	4.8
21.0	4.8
21.2	4.9
21.4	5.0
21.6	4.9
21.8	4.8
22.0	4.7
22.2	4.7
22.4	4.6
22.6	4.5
22.8	4.4
23.0	4.2
23.2	4.1
23.4	4.0
23.6	3.9
23.8	3.7
24.0	3.8
24.2	3.9
24.4	4.1
24.6	4.1
24.8	4.2
25.0	4.3
25.2	4.4
25.4	4.4
25.6	4.3
25.8	4.1
26.0	4.1
26.2	4.0
26.4	3.8
26.6	3.7
26.8	3.5
27.0	3.3
27.2	3.1
27.4	2.8
27.6	2.6
27.8	2.6
28.0	2.7
28.2	2.9
28.4	3.0
28.6	3.1
28.8	3.2
29.0	3.3
29.2	3.5
29.4	3.4
29.6	3.3
29.8	3.1
30.0	2.9
30.2	2.8
30.4	2.6
30.6	2.4
30.8	2.2
31.0	2.0
31.2	1.8
31.4	1.6
31.6	1.2
31.8	1.3
32.0	1.3
32.2	1.4
32.4	1.5
32.6	1.6
32.8	1.8
33.0	1.9
33.2	2.0
33.4	1.9
33.6	1.7
33.8	1.6
34.0	1.3
34.2	1.1
34.4	0.9
34.6	0.6
34.8	0.4
35.0	0.1
35.2	0.0
35.4	0.1
35.6	0.2
35.8	0.3
36.0	0.4
36.2	0.5
36.4	0.3
36.6	0.1
36.8	0.0
37.0	0.1
37.2	0.2
37.4	0.3
37.6	0.4
37.8	0.5
38.0	0.3
38.2	0.0
38.4	0.0
38.6	0.2
38.8	0.3
39.0	0.4
39.2	0.4
39.4	0.4
39.6	0.2
39.8	0.1
40.0	0.0
