# Supplementary material for: Liquid harvesting and transport on multiscaled curvatures
Source: Proc Natl Acad Sci U S A. 2020 Sep 8;117(38):23436–42. doi: 10.1073/pnas.2011935117 (PMC7519342; doi:10.1073/pnas.2011935117)
Supplement: Supplementary File [file pnas.2011935117.sd03.pdf]

Fig. 2*G* Individual condensate droplet weight with corresponding transport speed as a function of the condensation surface particular length on the dry and wet peristome surfaces.

Surface	State	Surface length, L, (mm)	Weight of water, w, (g)	Water transport velocity, v, (mm/s)
Retchet	Dry	0.41	4.30E-08	0.05
		0.41	5.70E-08	0.07
		0.41	5.30E-08	0.07
		0.41	7.10E-08	0.13
		0.41	6.90E-08	0.08
		0.46	5.20E-08	0.03
		0.46	6.50E-08	0.06
		0.46	6.90E-08	0.11
		0.46	8.40E-08	0.17
		0.46	7.20E-08	0.15
		0.48	5.70E-08	0.07
		0.48	5.20E-08	0.04
		0.48	6.50E-08	0.06
		0.48	6.90E-08	0.07
		0.48	7.30E-08	0.12
		0.50	7.50E-08	0.15
		0.50	7.20E-08	0.14
		0.50	5.90E-08	0.08
		0.50	6.20E-08	0.07
		0.50	6.80E-08	0.08
	Wet	0.41	3.60E-08	2.25
		0.41	5.20E-08	2.08
		0.41	5.50E-08	1.22
		0.41	6.30E-08	1.49
		0.41	4.90E-08	1.39
		0.46	4.40E-08	2.10
		0.46	5.50E-08	2.32
		0.46	5.90E-08	1.35
		0.46	6.70E-08	2.40
		0.46	6.20E-08	1.97
		0.48	4.70E-08	1.41
		0.48	3.20E-08	1.40
		0.48	4.50E-08	1.89
		0.48	3.90E-08	2.31
		0.48	4.30E-08	1.40
		0.50	5.50E-08	1.69
		0.50	5.20E-08	1.72
		0.50	2.90E-08	1.13
		0.50	4.20E-08	1.45
		0.50	3.80E-08	1.23
Concavity	Dry	0.79	6.30E-07	0.27
		0.79	6.70E-07	0.26
		0.79	7.70E-07	0.30
		0.79	6.10E-07	0.22
		0.84	6.80E-07	0.32
		0.84	7.30E-07	0.24
		0.84	7.50E-07	0.33
		0.84	7.90E-07	0.25
		0.87	8.10E-07	0.30
		0.87	8.40E-07	0.26
		0.87	7.20E-07	0.31
		0.87	9.10E-07	0.24
		0.91	8.50E-07	0.32
		0.91	7.30E-07	0.29
		0.91	7.90E-07	0.27
		0.91	9.50E-07	0.29
	Wet	0.79	6.20E-07	6.20
		0.79	5.70E-07	5.83
		0.79	6.30E-07	4.69
		0.79	5.20E-07	5.52
		0.84	5.30E-07	4.94
		0.84	6.60E-07	5.83
		0.84	6.90E-07	4.59
		0.84	7.10E-07	4.98
		0.87	6.30E-07	5.21
		0.87	7.10E-07	5.13
		0.87	6.70E-07	6.14
		0.87	6.30E-07	5.73
		0.91	7.20E-07	4.72
		0.91	6.60E-07	5.32
		0.91	5.40E-07	5.27
		0.91	6.50E-07	5.75
Arch	Dry	8.97	8.97	8.97
		9.33	9.33	9.33
		9.42	9.42	9.42
		9.84	9.84	9.84
		10.46	10.46	10.46
		10.59	10.59	10.59
		11.03	11.03	11.03
		11.36	11.36	11.36
	Wet	8.97	2.85E-02	184.24
		9.33	2.51E-02	175.35
		9.42	2.85E-02	192.74
		9.84	3.01E-02	198.23
		10.46	2.86E-02	183.51
		10.59	3.21E-02	193.40
		11.03	3.59E-02	188.84
		11.36	3.15E-02	183.24
