# Supplementary material for: Liquid harvesting and transport on multiscaled curvatures
Source: Proc Natl Acad Sci U S A. 2020 Sep 8;117(38):23436–42. doi: 10.1073/pnas.2011935117 (PMC7519342; doi:10.1073/pnas.2011935117)
Supplement: Supplementary File [file pnas.2011935117.sd04.pdf]

Fig. 3*D* Water harvest weight (w) versus time (t) for the water fog collected on the artificial peristome harvestor.

t (×10^3 s)	w_{Water} (g)
0.00	0.00
0.04	0.06
0.08	0.10
0.12	0.56
0.16	2.01
0.20	3.28
0.24	4.12
0.28	5.18
0.32	6.11
0.36	6.88
0.40	7.63
0.44	8.58
0.48	9.66
0.52	10.35
0.56	11.36
0.60	12.47
0.64	13.00
0.68	14.16
0.72	15.37
0.76	16.54
0.80	17.77
0.84	18.34
0.88	19.78
0.92	20.62
0.96	22.09
1.00	22.88
1.04	24.17
1.08	25.76
1.12	26.22
1.16	26.39
1.20	27.75
1.24	28.44
1.28	30.14
1.32	31.05
1.36	31.78
1.40	32.95
1.44	34.15
1.48	35.35
1.52	36.66
1.56	38.22
1.60	39.35
1.64	41.25
1.68	41.82
1.72	43.07
1.76	43.91
1.80	45.89
1.84	47.20
1.88	48.02
1.92	49.73
1.96	50.49
2.00	50.96
2.04	52.41
2.08	53.60
2.12	54.77
2.16	55.66
2.20	56.56
2.24	58.40
2.28	58.84
2.32	60.17
2.36	61.10
2.40	61.73
2.44	63.67
2.48	64.70
2.52	67.22
2.56	68.49
2.60	69.40
2.64	70.58
2.68	70.92
2.72	72.11
2.76	73.00
2.80	74.86
2.84	75.79
2.88	76.69
2.92	77.57
2.96	78.47
3.00	78.73
3.04	80.28
3.08	81.83
3.12	84.11
3.16	85.05
3.20	85.98
3.24	86.87
3.28	87.75
3.32	89.36
3.36	90.95
3.40	91.50
3.44	92.40
3.48	93.29
3.52	95.68
3.56	98.07
3.60	100.45
