# Supplementary material for: Liquid harvesting and transport on multiscaled curvatures
Source: Proc Natl Acad Sci U S A. 2020 Sep 8;117(38):23436–42. doi: 10.1073/pnas.2011935117 (PMC7519342; doi:10.1073/pnas.2011935117)
Supplement: Supplementary File [file pnas.2011935117.sd05.pdf]

Fig. 3*E* The water harvest weight (w) versus time (t) during the initial water condensation state.

t ($\times 10^2$ s)	w_{Water} (g)
0	0
0.04	0.01
0.08	0.02
0.12	0.03
0.16	0.03
0.20	0.04
0.24	0.04
0.28	0.04
0.32	0.05
0.36	0.05
0.40	0.06
0.44	0.06
0.48	0.06
0.50	0.06
0.52	0.07
0.56	0.07
0.60	0.07
0.64	0.08
0.68	0.08
0.72	0.08
0.76	0.09
0.80	0.10
0.84	0.10
0.88	0.11
0.92	0.13
0.96	0.14
1.00	0.15
1.04	0.27
1.08	0.38
1.12	0.42
1.16	0.45
1.20	0.56
1.24	0.69
1.28	0.76
1.32	0.88
1.36	1.00
1.40	1.13
1.44	1.26
1.48	1.46
1.50	1.63
1.52	1.75
1.56	1.88
1.60	2.01
1.64	2.15
1.68	2.27
1.72	2.40
1.76	2.53
1.80	2.66
1.84	2.79
1.88	2.91
1.92	3.03
1.96	3.15
2.00	3.28
