# Supplementary material for: Liquid harvesting and transport on multiscaled curvatures
Source: Proc Natl Acad Sci U S A. 2020 Sep 8;117(38):23436–42. doi: 10.1073/pnas.2011935117 (PMC7519342; doi:10.1073/pnas.2011935117)
Supplement: Supplementary File [file pnas.2011935117.sd06.pdf]

Fig. 4D The long term usage of artificial water harvestor and artificial oleogel harvestor.

	Harvest velocity (× 10^{-3} g cm^{-2} s^{-1})										
t (s)	water (40 °C)	water (60 °C)	water (80 °C)	isopropanol (25 °C)	isopropanol (40 °C)	kerosene (25 °C)	kerosene (40 °C)	gasoline (25 °C)	gasoline (40 °C)	glycol (25 °C)	glycol (40 °C)
7200	6.1 ± 0.17	6.3 ± 0.11	5.8 ± 0.13	4.2 ± 0.11	4.0 ± 0.11	3.7 ± 0.11	3.5 ± 0.09	3.4 ± 0.11	3.5 ± 0.10	2.5 ± 0.10	2.5 ± 0.12
36000	5.8 ± 0.14	6.1 ± 0.12	5.8 ± 0.17	4.1 ± 0.09	3.9 ± 0.09	3.9 ± 0.09	3.6 ± 0.09	3.3 ± 0.12	3.4 ± 0.11	2.3 ± 0.11	2.5 ± 0.10
108000	5.9 ± 0.09	6.1 ± 0.12	5.6 ± 0.21	4.2 ± 0.13	4.1 ± 0.12	3.8 ± 0.12	3.5 ± 0.10	3.4 ± 0.10	3.5 ± 0.09	2.4 ± 0.09	2.6 ± 0.11
216000	6.0 ± 0.13	6.0 ± 0.17	5.7 ± 0.15	4.0 ± 0.10	4.1 ± 0.10	3.6 ± 0.10	3.5 ± 0.10	3.4 ± 0.11	3.3 ± 0.12	2.4 ± 0.10	2.5 ± 0.12
432000	6.0 ± 0.20	6.1 ± 0.13	5.6 ± 0.19	4.1 ± 0.12	4.0 ± 0.11	3.6 ± 0.11	3.4 ± 0.09	3.3 ± 0.10	3.4 ± 0.11	2.3 ± 0.10	2.4 ± 0.11
