# Supplementary material for: Liquid harvesting and transport on multiscaled curvatures
Source: Proc Natl Acad Sci U S A. 2020 Sep 8;117(38):23436–42. doi: 10.1073/pnas.2011935117 (PMC7519342; doi:10.1073/pnas.2011935117)
Supplement: Supplementary File [file pnas.2011935117.sd07.pdf]

Fig. 4F Transport and harvest velocity of liquid condensation on different artificial structures.

Substrate	Transport velocity (mm s^{-1})	Harvest velocity (g cm^{-2} s^{-1})
Artificial silk	~ 0.013	~ 9.50×10^{-5}
	~ 0.35	~ 1.00×10^{-2}
Artificial spine	~ 0.02	~ 3.00×10^{-3}
	~ 0.03	~ 1.30×10^{-3}
	~ 116.70	~ 1.72×10^{-4}
	~ 8.75	~ $1.3.0 \times 10^{-4}$
	~ 0.33	~ 1.83×10^{-3}
	~ 0.80	~ 8.00×10^{-4}
Mesh	~ 0.40	~ 3.66×10^{-4}
	~ 0.63	~ 3.10×10^{-4}
	~ 4.00	~ 1.04×10^{-3}
	~ 5.00	~ 8.33×10^{-4}
	~ 140.00	~ 2.54×10^{-3}
Slippery surface	~ 0.15	~ 9.70×10^{-5}
	~ 0.03	~ 1.20×10^{-4}
Pitcher plant (This work)	~ 1,200.00	~ 6.80×10^{-2}
	~ 710.00	~ 4.20×10^{-2}
	~ 640.00	~ 3.70×10^{-2}
	~ 630.00	~ 3.40×10^{-2}
	~ 380.00	~ 2.50×10^{-2}
